# The insulin‐like growth factor system and the progression of renal complications of type 2 diabetes

**DOI:** 10.1111/dme.70318

**Published:** 2026-04-17

**Authors:** Adrian Heald, Yasitha lllangasekera, Andreas Matheou, Ram Prakash Narayanan, Stephen White, Hellena Habte‐Asres, Abigail Lay, Liliana Shalamanova, William Ollier, Graham A. Hitman, Ritwika Mallik, Philip A. Kalra, Martin Whyte, J. Martin Gibson

**Affiliations:** ^1^ University of Manchester Manchester UK; ^2^ Department of Diabetes and Endocrinology Salford Royal Hospital Salford UK; ^3^ Sheffield Hallam University Sheffield UK; ^4^ Faculty of Medicine University of Peradeniya Peradeniya Sri Lanka; ^5^ Diabetes Centre St Helens Hospital St Helens UK; ^6^ University of Newcastle Newcastle UK; ^7^ Kings College London London UK; ^8^ Manchester Metropolitan University Manchester UK; ^9^ Queen Mary University of London London UK; ^10^ Department of Renal Medicine Salford Royal Hospital Salford UK; ^11^ University of Surrey Guildford UK

**Keywords:** albuminuria, CKD, IGF, IGFBP, mortality, progression, type 2 diabetes

## Abstract

**Aims:**

Chronic kidney disease is associated with dysregulation of the insulin‐like growth factor (IGF) system. Alterations in IGF bioavailability, driven by IGF binding proteins (IGFBPs), may influence the decline in renal function. However, the longitudinal relationship between baseline IGF‐I, IGF‐II, IGFBPs and progression of kidney dysfunction remains unclear. We evaluated how IGF/IGFBP profile relates to longitudinal changes in urinary albumin/creatinine ratio (ACR) and serum creatinine level in people with type 2 diabetes (T2D).

**Methods:**

Individuals' measurements were performed in 436 individuals with T2D recruited from primary and secondary care (2002–2004), from the Salford and Manchester Integrated Care Record (GMCR), for laboratory and clinical data, as well as date of death. Baseline IGF/IGFBP profile was related to 2 primary outcomes: (i) change in albumin‐to‐creatinine ratio over time (urine ACR trend slope) and (ii) change in serum creatinine over time (creatinine trend slope). Trend slopes were derived for each participant, using repeated measures throughout follow‐up for up to 24 years. Associations between IGF axis proteins and outcomes were evaluated with multivariate regression models. Cox proportional hazards models were constructed to determine mortality risk associated with the IGF system proteins studied.

**Results:**

At baseline, 59.3% of participants were men. Age at baseline was 56.6 ± 9.4 years. Mean follow‐up duration was 17.4 ± 5.2 years.

IGF‐II and IGFBP‐3 were strongly correlated (Spearman rho = 0.80), and were therefore not included simultaneously in the multi‐variate linear regression model to avoid co‐linearity. Higher circulating IGFBP‐2 was independently associated with faster rate of ACR progression (normalised beta (Β) = 0.007, *p* = 0.002), while IGFBP‐1 showed an inverse association (*B* = −0.057, *p* = 0.009). IGF‐II also remained positively associated with ACR trend (*B* = 0.005, *p* = 0.013), whereas IGF‐I showed no association. IGFBP‐1 showed a significant inverse association with creatinine change when adjusted for effects of covariates (unstandardised *B* = −0.038, *p* = 0.04). Survival analysis revealed that higher IGFBP‐2 levels were independently associated with higher all‐cause mortality (hazard ratio [HR] per 1 standard deviation increase 1.306, 95% CI 1.151–1.483 *p* = 0.0001).

**Conclusion:**

This longitudinal study indicated that higher baseline IGF‐II, lower IGFBP‐1 and higher IGFBP‐2 are associated with a greater likelihood of albuminuria progression, independent of standard risk factors; lower IGBFP‐1 also associated with greater increase in serum creatinine. Higher circulating IGFBP‐2 level was also associated with relatively higher mortality. This study demonstrated the relevance of the IGF system to our understanding of renal complications in diabetes and strongly suggests that further exploration of mechanisms underlying these associations is merited.

**What’s new?**

- In this longitudinal study with up to 24 years of follow‐up of people with type 2 diabetes, we found that higher IGF‐II, lower IGFBP‐1 and higher IGFBP‐2 were independently associated with greater albuminuria progression over time.
- Lower circulating IGFBP‐1 was associated with a higher rate of increase of serum creatinine, with higher baseline IGFBP‐2 associated with increased all‐cause mortality.
- These findings highlight the relevance of measuring circulating IGF system biomarkers in relation to the potential prediction of the development of diabetes renal complications.

## 1 INTRODUCTION

Chronic kidney disease (CKD) is a major global public health problem and can result in end‐stage renal disease (ESRD), cardiovascular morbidity and premature death. CKD is a progressive disorder driven by known risk factors such as diabetes, hypertension and obesity, but also by more complex and less clear metabolic and hormonal disturbances.^1^ Among these, dysregulation of the growth hormone – insulin‐like growth factor and insulin‐like growth factor binding protein (GH–IGF–IGFBP) axis has emerged as a key feature of CKD pathophysiology.

We recently reported one of the longest follow‐up studies of people with type 2 diabetes (T2D) in relation to description of renal function change over time. Our results indicated that while urine albumin/creatinine ratio (ACR) increases exponentially over time, estimated glomerular filtration rate (eGFR) does so linearly.^2^

The presence of any features of CKD at recruitment to the study was associated with a markedly elevated 20+ year mortality rate^2^ with the highest mortality rate for those people with both an elevated ACR and an eGFR of <60 mL/min/1.73m^2^. In the same paper, a higher baseline high sensitivity C‐reactive protein (CRP) level was associated with faster decline in eGFR and greater fold increase in urine ACR over time.^3^

The IGF system, comprising IGF‐I and IGF‐II, their receptors and a family of both high‐ and low‐affinity IGFBPs, plays a central role in somatic growth, cellular metabolism and tissue repair.^4^ IGFs and IGFBPs are expressed in the kidney with diabetic nephropathy associated with dysregulation of the IGF system at the level of the glomerulus.^5^, ^6^ IGF‐I influences glomerular filtration, renal blood flow and nephron structure, while several IGFBPs, particularly IGFBP‐1, IGFBP‐2 and IGFBP‐3, modulate IGF bioavailability and exert IGF‐independent effects on apoptosis, inflammation and fibrosis. More recently, a higher baseline circulating IGFBP‐7 level was associated with the composite renal end point of sustained 40% reduction in estimated glomerular filtration rate, need for renal replacement therapy, or renal death in the CANVAS study.^7^

In CKD, circulating levels of IGFBPs are increased due to reduced renal clearance and altered proteolysis, leading to impaired IGF‐I bioavailability, despite normal or only modestly reduced total IGF‐I concentrations.^8^ These alterations contribute to acquired IGF resistance and may influence both renal disease progression and systemic complications. Most human studies to date have been cross‐sectional, and the long‐term prognostic significance of baseline IGF‐I and IGFBP levels for subsequent kidney function decline remains poorly defined.

We previously reported that in people with T2D, a high baseline IGFBP‐2 concentration was associated with a relatively more rapid decline in eGFR over an 8‐year period.^9^

This study investigates the association between baseline circulating IGF‐I, IGF‐II and IGFBP concentrations and longitudinal changes in kidney function, with the specific aim of clarifying the role of the IGF system in CKD progression and identifying potential biomarkers relating to renal decline.

## 2 METHODS

The study cohort consisted of adults with type 2 diabetes (T2D), recruited between 2002 and 2004 from Salford Royal NHS Foundation Trust clinics and local general practices and followed up for up to 24 years. Measurements were performed in 436 individuals with T2D. Ethical approval for the study was obtained from the Salford Research Ethics Committee (REC Reference 2001/156). Individuals who consented to participate donated a blood sample. Fasting serum and plasma blood samples were collected only at baseline and stored at −80°C after separation and aliquoting.

Baseline and follow‐up clinical and laboratory data were obtained through the Salford Integrated Record^10^ and the Greater Manchester Care Record.^11^ The only inclusion criterion was a diagnosis of T2D at recruitment; no exclusion criteria were applied. Participants remained under follow‐up until death, ‘lost to follow‐up’ or censoring. Salford is a geographically stable population with a low emigration rate, and changes in population numbers over time are therefore likely primarily to reflect mortality.

CKD was defined as a urine albumin: creatinine ratio (ACR) >3 mg/mmol and/or estimated glomerular filtration rate (eGFR) <60 mL/min/1.73m^2^.

### 2.1 Laboratory methods

Measurements of serum IGF‐I, lithium heparin plasma IGF‐II, lithium heparin plasma IGFBP‐1, lithium heparin plasma IGFBP‐2 and serum IGFBP‐3 were taken from the blood sample collected at recruitment. IGF‐I was measured using an IMMULITE 1000 immunoassay (Siemens, Camberley, UK) with an analytical sensitivity of 20 ng/mL and within and inter‐assay coefficients of variation (CV) of 4.5 and 8.4% respectively for IGF‐I between 20 and 350 ng/mL. IGF‐II was measured using an ELISA developed using antibodies that have been previously reported.^12^ The analytical sensitivity of the assay was 10 ng/mL. The intra‐assay and inter‐assay CV was 6% and 10% respectively between 10 and 1000 ng/mL. IGFBP‐1 was measured using a locally developed assay that recognised all phosphor‐forms of IGFBP‐1 and has been reported in a previous study.^13^ This assay had inter‐assay and intra‐assay CV was 15% and 9% between 5 and 150 ng/mL, respectively, and the assay had an analytical sensitivity of 0.1 ng/mL. IGFBP‐2 measurements were performed using the commercial RayBio Human IGFBP‐2 ELISA kit (Ray Biotech, Inc., Norcross, GA, USA) with a manufacturer reported sensitivity of 20 pg/mL. The intra‐assay CV for this assay was 10% and the intra‐assay CV was 12% between 10 and 500 ng/mL. Plasma IGFBP‐3 was measured using the IMMULITE 1000 immunoassay that had an analytical sensitivity of 0.1 mcg/mL. The intra‐assay CV for this assay was 6% and the inter‐assay CV was 10% between 2 and 30 mcg/mL. High sensitivity C‐reactive protein (hsCRP) was also measured on the IMMULITE 1000 immunoassay platform. The intra‐assay CV for this assay was 3% and the inter‐assay CV was 7% between 0.1 and 30 mcg/mL.

### 2.2 Statistical analysis

Continuous variables are presented as mean ± standard deviation and categorical variables as counts and percentages. Associations between circulating IGFs, IGFBPs, and longitudinal renal outcomes were examined using linear regression models.

Two primary outcomes were analysed: (i) change in urinary ACR over time (urine ACR trend slope) and (ii) change in serum creatinine over time (creatinine trend slope). Trend slopes were derived for each participant using repeated measures over follow‐up.

Univariate and multivariate linear regression analyses were performed to assess associations between IGF‐I, IGF‐II, IGFBP‐1, IGFBP‐2, IGFBP‐3 and renal outcome slopes. Multivariate models were adjusted for potential covariates selected on clinical relevance, including age, sex, body mass index trend, diabetes duration, smoking status, systolic and diastolic blood pressure trends, glycaemic control (HbA1c trend), baseline total cholesterol and C‐reactive protein. There was at least one value every 2 years up to last follow‐up. All covariates were entered simultaneously.

The ACR trend for a given subject is the ‘average per year change in ACR’ within the overall study period. It is the calculated slope (B) of the best fit regression line through all ACR values of a given subject. So, a positive value denotes an average increase in ACR per year and a negative a reduction. The trend slope is therefore a more dynamic measure of change as opposed to just taking the average of all available ACR values for a given subject. The number of ACR values available for each subject varied and whatever available values were used to calculate the trend slope.

Given strong correlations between certain IGF system markers, collinearity was assessed using correlation coefficients. Highly correlated predictors were not included together in the same model to avoid collinearity and to preserve interpretability of regression coefficients. Cox proportional hazards models were constructed to determine mortality risk associated with IGF system proteins, adjusted for demographic, lifestyle and other metabolic factors. Regression coefficients are reported as unstandardised (B) and standardised (β) estimates with corresponding standard errors and *p* values. IGFBP‐1 was log transformed for visualisation due to its highly skewed distribution.

Analyses were performed using IBM SPSS Statistics (ver. 25), Armonk, New York, USA.

## 3 RESULTS

The baseline characteristics of the study population are shown in Table 1.

**TABLE 1:** Baseline characteristics of study population.

Characteristic	Mean SD or *n* (%)
Age (years)	56.3 ± 9.4
Male %	59.3
BMI (kg/m^2^)	31.4 ± 5.9
Established CKD Based on eGFR<60 mL/min/1.73m^2^	514/612 (84%)
Based on ACR >3 mg/mmol	77/340 (22.6%)
Diabetes duration (years)	20.9 ± 6.9
Smoking status	
Smoker	90 (12.5%)
Ex‐smoker	298 (41.5%)
Non‐smoker	323 (45%)
SBP (mmHg)	139.5 ± 17.9
DBP (mmHg)	75.6 ± 11.0
Cholesterol (mmol/L)	5.1 ± 1.6
HbA1c (%)	8.1 ± 1.7
Creatinine	90.5 ± 25.3
ACR	4.8 ± 14.0
eGFR	77.5 ± 19.0
IGF‐I (ng/mL)	136.4 ± 57.2
IGF‐II (ng/mL)	600.1 ± 208.1
IGFBP‐1 (ng/mL)	19.5 ± 18.6
IGFBP‐2 (ng/mL)	318.9 ± 188.9
IGFBP‐3 (mcg/mL)	4.5 ± 1.5
hsCRP (mg/L)	7.3 ± 11.5

Abbreviations: BMI, Body mass index; DBP, diastolic blood pressure; HbA1c, glycated haemoglobin; hsCRP, high sensitivity C‐reactive protein; IGF‐I, insulin growth factor‐1; IGF‐II, insulin‐like growth factor‐II; IGFBP‐1, insulin‐like growth factor binding protein‐1; IGFBP‐2, insulin‐like growth factor binding protein‐2; IGFBP‐3, insulin‐like growth factor binding protein‐3; SBP, systolic blood pressure.

At baseline, 84% and 22.6% of the study population had CKD based on an eGFR >60 or ACR >3. Nine years into follow up, 491/661 (74.3%) and 103/346 (29.8%) had CKD based on eGFR and ACR respectively. The results for the univariate regression analysis predicting ACR trend from each IGF ligand and IGF binding protein, are shown in Table 2. Except for IGF‐I, all other factors tested were shown to be positive predictors of ACR progression.

**TABLE 2:** Univariate regression of ACR change by IGF ligand or IGF binding protein. The ACR trend—the dependent variable in the regression model—is made up of both positive and negative values. It has a range of 96 (min = − 25.38, maximum = +71.5).

IGF ligand/binding protein	*B* (unstandardised)	Standard error of *B*	Standardised *β*	*p* value
IGF‐I	0.006	0.006	0.052	0.316
IGF‐II	0.005	0.002	0.152	0.003
IGFBP‐1	−0.040	0.020	−0.103	0.045
IGFBP‐2	0.004	0.002	0.109	0.034
IGFBP‐3	0.544	0.242	0.115	0.025

Abbreviations: IGF‐I, insulin growth factor‐1; IGF‐II, insulin‐like growth factor‐II; IGFBP‐1, insulin‐like growth factor binding protein‐1; IGFBP‐2, insulin‐like growth factor binding protein‐2; IGFBP‐3, insulin‐like growth factor binding protein‐3.

The multivariate regression predicting ACR progression over the 24‐year follow up period is shown in Table 3. IGF‐II and IGFBP‐3 were strongly correlated (Spearman rho = 0.80) and were therefore not included simultaneously into the model to avoid co‐linearity. Whilst IGFBP‐1 and IGFBP‐2 were also correlated, this was much weaker (Spearman rho = 0.30) and therefore included in the model. As seen in Table 3, baseline IGF‐II, IGFBP‐1 and IGFBP‐2 levels were significant independent predictors of urine ACR increase. The predictive strength of these factors remained even when unadjusted for covariates. Higher IGFBP‐2 was independently associated with more rapid ACR progression (*B* = 0.007, *p* = 0.002), while IGFBP‐1 showed an inverse association (*B* = −0.057, *p* = 0.009). IGF‐II also remained positively associated with ACR trend (*B* = 0.005, *p* = 0.013), whereas IGF‐I showed no association.

**TABLE 3:** Multivariate regression analysis of factors associated with ACR change.

Predictor	*B* (unstandardised)	Standard error	*Β* (standardised)	*p* value
Constant	1.900	4.049	—	0.639
IGF‐I	−0.001	0.008	−0.005	0.935
IGF‐II	0.005	0.002	0.155	0.013
IGFBP‐2	0.007	0.002	0.181	0.002
IGFBP‐1	−0.057	0.022	−0.146	0.009
Age (final)	−0.044	0.041	−0.058	0.283
Sex	−0.177	0.862	−0.012	0.838
BMI trend	−0.298	0.734	−0.021	0.685
Diabetes duration	−0.051	0.056	−0.050	0.362
Smoking	0.733	0.587	0.067	0.213
SBP trend	−0.360	0.239	−0.093	0.133
Cholesterol (baseline)	−0.020	0.231	−0.005	0.929
HbA1c trend	−2.563	2.347	−0.059	0.276
DBP trend	0.486	0.407	0.073	0.234
hsCRP	0.043	0.035	0.068	0.219

Abbreviations: BMI, Body mass index, DBP, diastolic blood pressure, HbA1c, glycated haemoglobin, hsCRP, high sensitivity C‐reactive protein, IGF‐I, insulin growth factor‐1, IGF‐II, insulin‐like growth factor‐II, IGFBP‐1, insulin‐like growth factor binding protein‐1, IGFBP‐2, insulin‐like growth factor binding protein‐2, IGFBP‐3, insulin‐like growth factor binding protein‐3, SBP, systolic blood pressure.

The associations between IGF‐II, IGFBP‐1 and IGFBP‐2 with ACR is shown in Figure 1a–c. IGFBP‐1 is log transformed because its distribution is highly skewed.

**FIGURE 1:**
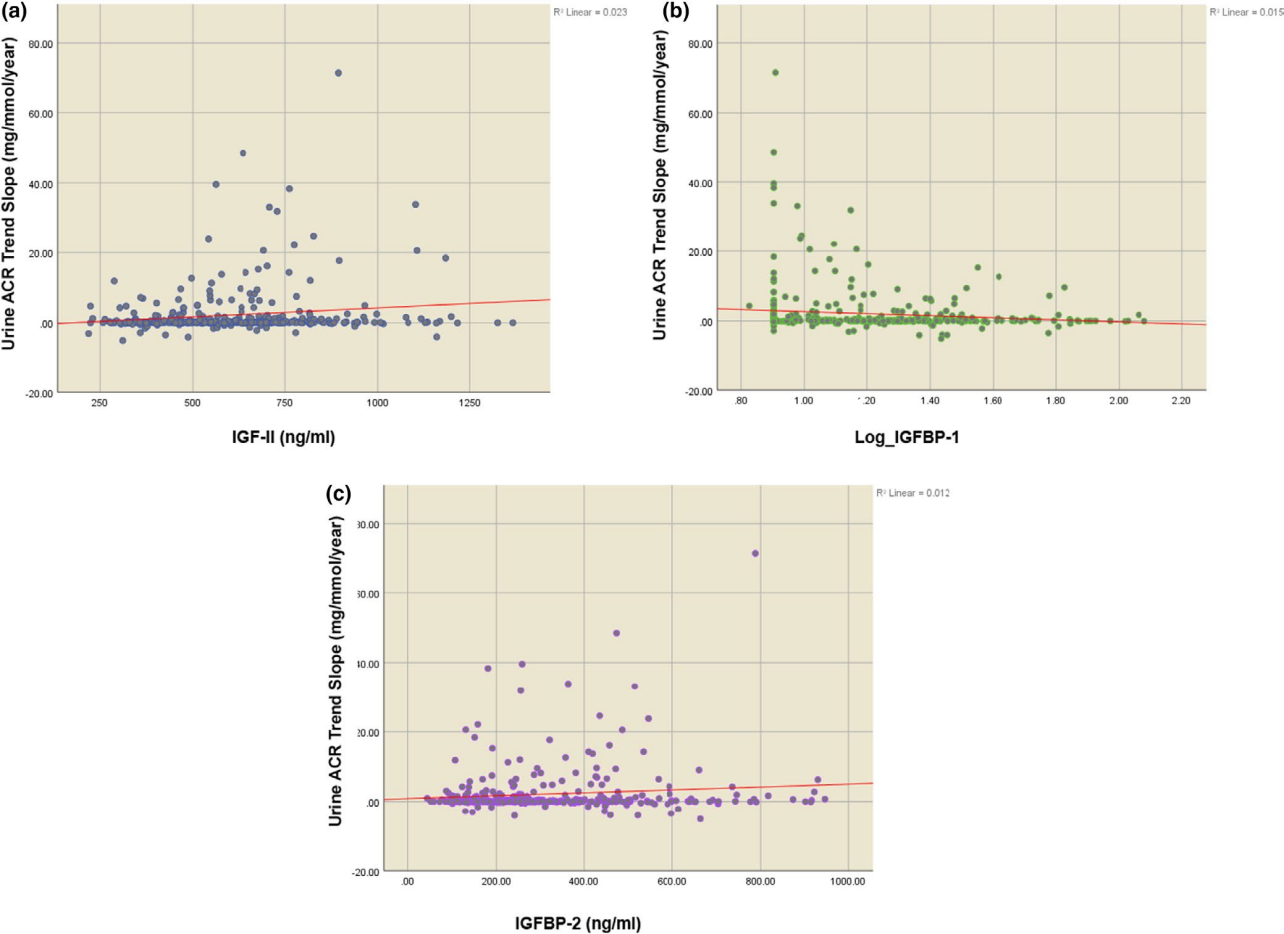
(a) Urinary ACR trend slope by baseline IGF‐II concentration. The red line is the univariate regression line. (b) Urinary ACR trend slope by baseline log IGFBP‐1. The red line is the univariate regression line. (c) Urinary ACR trend slope by baseline IGFBP‐2. The red line is the univariate regression line.

The multivariate regression predicting serum creatinine trends by IGFBPs are shown in Table 4. IGFBP‐1 showed a significant inverse association with greater creatinine change when adjusted for effects of covariates (Unstandardised *B* = −0.038, *p* = 0.04). IGFBP‐2 demonstrated a ‘borderline’ association that did not reach statistical significance.

**TABLE 4:** Multivariate regression analysis of factors associated with serum creatinine change.

Variable	*B* (unstandardised)	Std. error	*β* (standardised)	*p* value
Constant	−0.225	3.366		0.947
IGF1	0.004	0.006	0.042	0.489
IGF2	−0.001	0.002	−0.037	0.554
IGFBP‐2	0.003	0.002	0.109	0.054
IGFBP‐1	−0.038	0.018	−0.114	0.040
Age	0.003	0.034	0.004	0.939
Sex	1.062	0.720	0.086	0.141
BMI trend	0.071	0.612	0.006	0.908
Diabetes Duration	0.044	0.047	0.051	0.347
hsCRP	0.031	0.029	0.059	0.280
Smoking	0.323	0.490	0.035	0.510
SBP trend	−0.682	0.163	−0.249	<0.001
DBP trend	0.695	0.323	0.128	0.032
Baseline cholesterol	0.030	0.193	0.008	0.879
HbA1c trend	−0.085	1.947	−0.002	0.965

Abbreviations: BMI, Body mass index, DBP, diastolic blood pressure, HbA1c, glycated haemoglobin, hsCRP, high sensitivity C‐reactive protein, IGF‐I, insulin growth factor‐1, IGF‐II, insulin‐like growth factor‐II, IGFBP‐1, insulin‐like growth factor binding protein‐1, IGFBP‐2, insulin‐like growth factor binding protein‐2, IGFBP‐3, insulin‐like growth factor binding protein‐3, SBP, systolic blood pressure.

The covariate‐adjusted Cox proportional hazards models for mortality risk associations with IGF‐II, IGFBP‐1 and IGFBP‐2 are shown in Table 5.

**TABLE 5:** Cox proportional hazard ratios for mortality with IGF proteins adjusted for covariates.

Variable	Hazard ratio (HR)	95% CI lower	95% CI upper	*p* value
IGF‐I (per SD increase)	1.277	1.098	1.485	0.002
IGF‐II (per SD increase)	0.794	0.675	0.934	0.005
IGFBP1 (per SD increase)	0.996	0.873	1.137	0.952
IGFBP2 (per SD increase)	1.332	1.172	1.515	0.000
Smoking	1.295	1.060	1.582	0.011
Sex	0.729	0.552	0.962	0.026
Total cholesterol	1.042	0.937	1.159	0.447
BMITrend	0.922	0.688	1.237	0.589
eGFRTrend	0.880	0.799	0.969	0.010
ACRTrend	1.034	1.017	1.051	0.000
SBPTrend	0.908	0.819	1.006	0.065
DBPTrend	1.120	0.941	1.333	0.202
Diabetes duration	0.983	0.962	1.003	0.096
HbA1c trend	0.135	0.053	0.342	0.000

Abbreviations: BMI, body mass index; DBP, diastolic blood pressure; HbA1c, glycated haemoglobin; hsCRP, high sensitivity C‐reactive protein; IGF‐I, insulin growth factor‐1; IGF‐II, insulin‐like growth factor‐II; IGFBP‐1, insulin‐like growth factor binding protein‐1; IGFBP‐2, insulin‐like growth factor binding protein‐2; IGFBP‐3, insulin‐like growth factor binding protein‐3; SBP, systolic blood pressure.

Greater circulating IGF‐I and IGFBP‐2 levels were independently associated with higher all‐cause mortality: hazard ratio [HR] per 1 SD increase in IGF‐I was 1.277 (95% CI 1.098–1.485; *p* = 0.002) and IGFBP‐2 was 1.332 (95% CI 1.172–1.515; *p* = 0.000). In contrast, greater IGF‐II levels were associated with a reduction in all‐cause mortality risk 0.794 (95% CI 0.675–0.934; *p* = 0.005). Among clinical covariates, current smoking and increasing ACR trend was associated with increased mortality risk whilst increasing eGFR trend and HbA1c were showed an inverse association with mortality risk. Diabetes duration, BMI trend, systolic and diastolic blood pressure trends and baseline cholesterol were not independently associated with mortality risk in the adjusted model.

A Kaplan Meier survival curve by quartiles of baseline IGFBP‐2 is shown in Figure 2. This demonstrates a lower survival, up to 23 years follow‐up, by increasing IGFBP‐2 at baseline. Survival analysis revealed that higher IGFBP‐2 levels (approximately 186 ng/mL, 59% of the mean) were independently associated with higher all‐cause mortality (hazard ratio (HR) per 1 standard deviation increase in IGFBP‐2 was 1.306, 95% CI 1.15–1.48, *p* = 0.0001).

**FIGURE 2:**
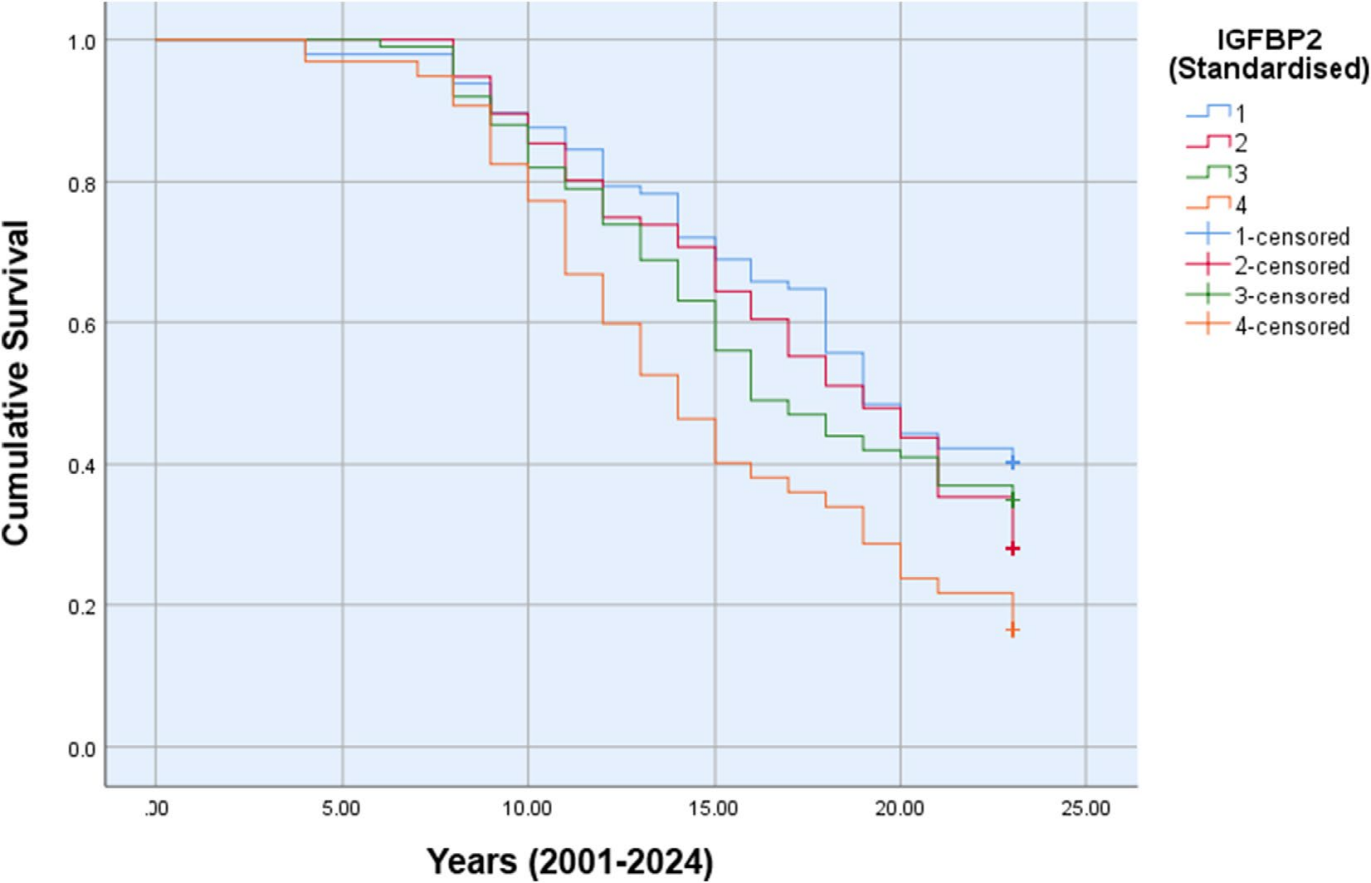
Kaplan Meier survival function for death vs. increasing quartiles of IGFBP‐2.

## 4 DISCUSSION

In this longitudinal study of individuals with T2D, over 24 years of follow up, we demonstrate that components of the IGF system at baseline are associated with progression of albuminuria and mortality. Specifically, higher baseline concentrations of IGF‐II and IGFBP‐2 and lower concentrations of IGFBP‐1, were independently associated with faster increases in urinary ACR over time, while lower circulating IGFBP‐1 was associated with a greater increase over time in serum creatinine in univariate and multivariate analysis. In addition, higher baseline IGFBP‐2 was independently associated with increased all‐cause mortality.

Among the IGF system components examined, IGFBP‐2 emerged a predictor of both increase in ACR and higher mortality. This was even after adjustment for traditional risk factors and longitudinal changes in glycaemic control, blood pressure, kidney function and albuminuria. We would suggest that ACR is a more sensitive marker of renal damage in type two diabetes than is serum creatinine, which has a multifactorial aetiology including renovascular disease in relation to its increase.

The participant sampling mostly occurred in general practices in Salford and was therefore representative of the overall group of individuals with T2D living in that area at the time when recruitment was undertaken. The demographic profile is similar to all people with T2D in Salford as described in a recent paper.^14^

IGFBP‐2 is the second most abundant circulating IGFBP and has a greater affinity for IGF‐II than for IGF‐I.^15^ In addition to regulating IGF‐I bioavailability, IGFBP‐2 exerts both IGF‐dependent and IGF‐independent biological effects.^16^, ^17^, ^18^ We previously reported that a high IGFBP‐2 concentration at baseline was associated with a decreased eGFR over an 8‐year period.^9^ In the kidney, IGFBP‐2 expression has been demonstrated in the renal glomerulus, thin limbs of the loop of Henle, and medullary collecting ducts and may play an important role in renal podocyte development and maturation.^19^, ^20^, ^21^, ^22^ IGFBP‐2 is abundantly expressed in the glomerulus, similar to the other IGFBPs: IGFBP‐1, IGFBP‐4 and IGFBP‐5.^22^

Several studies have reported increased IGFBP‐2 concentrations in chronic renal failure,^23^ and have been associated with inflammation, protein‐energy wasting, and adverse metabolic profiles, all of which may contribute to increased mortality risk. This apparent paradox may be explained by differences between local intra‐renal IGFBP‐2 activity and circulating IGFBP‐2 concentrations. While IGFBP‐2 expression within the kidney may support podocyte biology, elevated serum IGFBP‐2 likely reflects systemic metabolic or inflammatory stress and greater disease severity.

The findings we report of adverse outcomes with raised IGFBP‐2 are consistent with previous reports linking elevated IGFBP‐2 to poorer health outcomes in people with T2D. In a 20‐year follow‐up study in older individuals with T2D, van den Beld et al reported a significant relation between higher IGFBP‐2 concentrations in serial measurements and an increased risk for death, with a unit increase of log2(IGFBP‐2) increasing the log hazard by 1.19 (95% CI, 0.70–1.63, *p* < 0.001) when adjustment was made for insulin sensitivity.^24^

Whether IGFBP‐2 plays a direct pathogenic role in glomerular injury or primarily reflects underlying metabolic and inflammatory stress cannot be determined from this longitudinal study. Such exploration will require detailed transcriptomics and pre‐clinical studies. However, strong and independent associations of IGFBP‐2 with both renal and survival outcomes support its potential utility as a prognostic biomarker.

In contrast to IGFBP‐2, lower circulating IGFBP‐1, whose production at the liver is heavily downregulated by insulin^25^, ^26^ was associated with a more rapid rate of ACR progression and creatinine increase. This relation aligns with emerging experimental and human data suggesting a protective role for IGFBP‐1 within the glomerulus. Data from the Pima diabetic kidney disease cohort and from the Nephroseq database demonstrated a significant reduction in glomerular IGFBP‐1 in the early stages of human T2D diabetic kidney disease.^27^

In the glomerulus, IGFBP‐1 is predominantly expressed in podocytes and controlled by phosphoinositide 3‐kinase (PI3K)‐fork head box O1 (FoxO1) activity. In vitro, IGFBP‐1 signalled to podocytes via β1‐integrins, increasing podocyte motility, adhesion, electrical resistance across the adhesive cell layer and cell viability.^28^ Thus, at a local level in the kidney, IGFBP‐1 may have a protective role in reducing the cellular processes that lead to renal damage in T2D. Genetic studies further support this concept. We have previously shown that single nucleotide polymorphisms (SNPs) in IGFBP‐1 were associated with a reduced prevalence of diabetic nephropathy. Haplotype analysis revealed that 97% of the genetic variation for circulating serum IGFBP‐1 in the population sample could be accounted for using two of the “reno‐protective” SNPs^29^ Together, these data suggest that higher circulating IGFBP‐1 may reflect preserved podocyte function or more favourable insulin‐sensitive metabolic states, potentially mitigating glomerular permeability and albumin leakage.

In the diabetic rat kidney, IGFBP levels, particularly IGFBP‐1, also increase early in the disease process^30^, ^31^ and it has been postulated that this could contribute to IGF‐I accumulation. IGF‐I and IGF‐II receptor levels also are increased in animal models of diabetic nephropathy.^32^ In patients with diabetic nephropathy, urinary IGFBP‐3 protease activity correlates with the degree of albuminuria, and it was postulated that this could contribute to kidney damage due to increased local IGF‐I activity.^33^

IGF‐II is a multifunctional polypeptide hormone that serves important roles in embryonic development, metabolic regulation, and disease pathogenesis,^34^ IGF‐II mRNA is widely expressed in renal tissue.^34^ Transgenic mice overexpressing IGF‐II demonstrate increased renal size.^35^ A study in STZ‐induced diabetes rats reported an increase in serum and kidney IGF‐II concentrations after the onset of diabetes, but without an increase in renal IGF‐II mRNA expression.^36^ This would suggest that the increased renal IGF‐II concentration was derived from the systemic circulation rather than produced within the kidney itself. Basal IGF‐II also contributes to renal homeostasis by supporting cellular repair and metabolic balance.^37^, ^38^, ^39^, ^40^ IGF‐II expression is upregulated in diabetic kidneys.^41^ Through activation of the IGF‐I receptor and the insulin receptor, IGF‐II induces abnormal glomerular cell proliferation and promotes extracellular matrix deposition, resulting in glomerulosclerosis and compromise of glomerular filtration barrier integrity.^42^ These changes not only impair renal structure and function but also accelerate the progression of diabetic kidney disease.^43^, ^44^ Our findings support a role for IGF‐II in promoting glomerular injury and albuminuria progression in diabetes.

In the CREDENCE study, higher IGF‐I levels (for a given age) were associated with a greater risk of developing renal and all‐cause mortality events. 3 years of therapy with canagliflozin did not significantly change IGF‐I and IGFBP‐3 concentrations.^45^ We did not see the same relation here between baseline circulating IGF‐I levels and development of diabetic kidney disease or with all cause mortality. The difference may relate to our study being of much longer duration and being an observational study vs. CREDENCE as a clinical trial, as well as the complexities of IGF system biology and the limitations of relating serum levels of hormonal biomarkers to the measured consequences of diabetes tissue complications.

In a study that compared individuals with non‐diabetic chronic kidney disease subjects with healthy controls, Frystyk et al.^46^ reported significant increases in total serum IGF‐II as well as IGFBP‐1 and IGFBP‐2 in those with nephropathy. The higher IGFBP‐1 levels reported (rather than lower as we found) may relate to the fact that the study was in non‐diabetic individuals (unliked T2D individuals as here) and was cross‐sectional in its design.

The dissociation between albuminuria progression and loss of filtration function is increasingly recognised in diabetic kidney disease^47^ and suggests that these outcomes reflect distinct, though overlapping, pathophysiological processes. Albuminuria is a sensitive marker of glomerular endothelial and podocyte injury and often precedes measurable declines in eGFR / rise in serum creatinine. Our findings imply that IGF system alterations may preferentially influence glomerular permeability and microvascular injury rather than nephron loss or tubular dysfunction that drives eGFR decline.

Insulin‐like growth factor binding‐protein 7 (IGFBP‐7) is expressed in renal tubular cells and is elevated in both blood and urine during the early stages of diabetes. A recent study found that tubule‐ and glomerular‐specific IGFBP‐7 promotes DKD, with tubular‐derived IGFBP‐7 disrupting the renal microenvironment. IGFBP‐7 impairs mitochondrial bioenergetics in tubular cells, with resultant lipid accumulation, cell cycle arrest, interstitial inflammation, fibrosis and glomerulosclerosis. IGFBP‐7 was proposed as a promising therapeutic target for DKD.^48^

The association between higher IGFBP‐2 and mortality observed in this study further underscores the systemic relevance of IGF system dysregulation. Importantly, this association persisted after adjustment for creatinine and ACR trajectories, suggesting that IGFBP‐2 captures risk beyond kidney disease severity alone. Whilst our study is not the first to describe an association between higher circulating IGFBP‐2 and increased mortality, our study extends these findings to a population with T2D. IGFBP‐2 may therefore reflect broader processes such as inflammation, sarcopenia, or cardiovascular vulnerability, which contribute to long‐term mortality in diabetes.

Our findings can be viewed in the context of previously reported long‐term outcomes in this cohort, in which a high proportion of individuals with elevated baseline ACR died during extended follow‐up.^2^, ^3^ This highlights the clinical relevance of identifying biomarkers associated with albuminuria progression and adverse outcomes.

Our study has several strengths, including its long duration of follow‐up, repeated measures allowing slope‐based analyses of kidney outcomes, and simultaneous assessment of renal and mortality endpoints. However, we did not account for the effects of angiotensin converting enzyme (ACE)‐inhibitors, angiotensin receptor blockers (ARBs), or other concomitant medications. These are known to alter creatinine slopes and to moderate changes in urine ACR. IGF ligands and binding proteins were measured at a single (baseline) time point, preventing assessment of temporal changes in relation to disease progression. Residual confounding and survivor bias are also a possibility, and urinary IGF system components were not measured. Furthermore IGFBPs 4–7 were not analysed here. This is of relevance given that in the CANVAS study in T2D, a higher baseline circulating IGFBP‐7 level was associated with the composite renal end point of sustained 40% reduction in estimated glomerular filtration rate, need for renal replacement therapy, or renal death in the CANVAS study.^7^

In conclusion, in this longitudinal study with up to 24 years of follow‐up, we found that higher IGF‐II, lower IGFBP‐1, and higher IGFBP‐2 were independently associated with greater albuminuria progression over time and in the case of IGFBP‐1 with a higher rate of increase of serum creatinine, with higher baseline IGFBP‐2 associated with increased all‐cause mortality. These findings highlight the relevance of the IGF system to our understanding of renal complications in diabetes and emphasise the importance of distinguishing biomarkers of glomerular injury from those of filtration loss. Further studies are now warranted to clarify the mechanistic role of IGF system components and to evaluate their potential utility in risk prediction and therapeutic targeting.

## AUTHOR CONTRIBUTIONS

AH, RPN and JMG conceived the study and designed the protocol in consultation with PK. AM and YI performed data processing and data analysis in consultation with RPN, JMG, WO, PK and AH. SW, LS, AL and HHA provided scientific context together with JMG, GH, WO, RM, MBW and PK who also provided senior review. All authors reviewed the manuscript during its development, approved the final version and agreed to be accountable for all aspects of the work.

## FUNDING INFORMATION

No external funding was received for the work reported here.

## CONFLICT OF INTEREST STATEMENT

None of the authors have any conflicts of interest to declare.

## Data Availability

The data that supports the findings of the study are available on reasonable request.
